# Preliminary Exploration of a New Therapy for Interstitial Cystitis/Bladder Pain Syndrome: Botulinum Toxin A Combined with Sapylin

**DOI:** 10.3390/toxins14120832

**Published:** 2022-11-30

**Authors:** Wenshuang Li, Zhenming Zheng, Kaiqun Ma, Caixia Zhang, Kuiqing Li, Paierhati Tayier, Yousheng Yao

**Affiliations:** 1 Department of Urology, Sun Yat-Sen Memorial Hospital, Sun Yat-Sen University, Guangzhou 510120, China; 2 Guangdong Provincial Key Laboratory of Malignant Tumor Epigenetics and Gene Regulation, Sun Yat-Sen Memorial Hospital, Sun Yat-Sen University, Guangzhou 510120, China; 3 Guangdong Provincial Clinical Research Center for Urological Diseases, Guangzhou 510120, China; 4 Department of Urology, The Eighth Affiliated Hospital of Sun Yat-Sen University, Shenzhen 518033, China; 5 Department of Urology, Shantou Central Hospital, Shantou 515031, China

**Keywords:** bladder pain syndrome, botulinum toxin A, interstitial cystitis, Sapylin

## Abstract

Interstitial cystitis/bladder pain syndrome (IC/BPS) is an intractable disease without long-term effective therapy. This study aims to evaluate the efficacy and safety of botulinum toxin A (BoNT/A) plus Sapylin, which might modulate the immune response of the bladder in the treatment of IC/BPS patients. We retrospectively investigated the clinical outcomes among 34 patients who accepted repeated Sapylin instillations after 200 U of BoNT/A submucosally injected into bladder walls (Mix group) and 28 patients who received BoNT/A alone (Control group). Each of the bladder walls (left, right, anterior and posterior) was injected six times with 8 U of BoNT/A per injection. The primary outcome measure was the global response assessment. The results showed that at 6 months post-injection, the response rate in the Mix group was remarkably higher than that in the Control group (58.8% vs. 28.6%, *p* < 0.05). The mean effective duration of the responders in the Mix group was apparently better than that in the Control group (27.5 (range 0–89) vs. 4.9 (range 0–11) months, *p* < 0.05). None of the patients experienced serious adverse events. In conclusion, repeated intravesical instillations of Sapylin after BoNT/A injection can produce significantly better clinical outcomes than BoNT/A alone in IC/PBS patients.

## 1. Introduction

Interstitial cystitis/bladder pain syndrome (IC/BPS) is a chronic disease with suprapubic pain or discomfort related to bladder filling, urinary frequency and urgency, resulting in a serious impairment of quality of life [1]. However, the current treatments for IC/BPS struggle to maintain long-term efficacy and the symptoms of IC/BPS are prone to recurrence because of its obscure etiology and pathogenesis [2].

It is proposed that an injured urothelium and neural hypersensitivity of the bladder might exacerbate chronic inflammation and immune responses in IC/BPS patients, causing persistent bladder pain and urinary frequency [3,4]. In fact, bladder pain or discomfort often drives urinary frequency and nocturia [5]. Botulinum toxin A (BoNT/A) might decrease the neural hypersensitivity of the bladder to relieve bladder pain and urination frequency and urgency [6]. However, the average effective duration of BoNT/A injection was only about 6 months, so repeated injection was required [7]. Therefore, a new therapy to extend the response duration is needed to reduce the burden on patients and health systems.

The immune and inflammatory responses might play a crucial role in the pathogenesis of IC/BPS [1]. Many researchers have shown that inflammatory factors, such as interleukin-6 and tumor necrosis factor-alpha (TNF-α), are significantly increased in the bladder tissue of IC/BPS patients [8,9,10]. In addition, Bosch’s research demonstrated that subcutaneous certolizumab pegol, an anti-TNF-α agent, significantly improved patients’ symptoms compared with placebo therapy [11]. Later, Mishra et al. pointed out that an intravesical instillation of tacrolimus had a significant effect on the treatment of IC/BPS by inhibiting the immune response [12]. These conditions show that immunotherapy for IC/BPS is a reasonable option.

Sapylin (OK-432) is a lyophilized preparation made from a low-virulence strain (Su) of Streptococcus pyogenes (group A) incubated with penicillin [13], which is successfully used as an immunotherapeutic agent in many malignant cancers [14,15]. In addition, many researchers found that Sapylin also had immunotherapeutic effects on bladder cancer, creating the possibility for it to treat bladder diseases [16,17,18]. In an animal study, researchers found that Sapylin might accelerate wound closure and promote angiogenesis, collagen synthesis and the remodeling process to improve wound healing and reduce seroma formation [19]. These promising results seemed to indicate a possibility that Sapylin might be used as an immunotherapeutic agent in IC/BPS by repairing injured urothelium.

At present, there are many therapeutic methods for relieving IC/BPS symptoms but these are symptomatic and their efficacy is not long lasting. As a consequence, most IC/BPS patients inevitably suffer again and continue to wait for an innovative formulation. Luckily, we found a new treatment scheme (repeated intravesical instillations of Sapylin after BoNT/A injection) that could more persistently improve the symptoms of IC/BPS patients. In this study, we would like to summarize and share the results of our preliminary exploration.

## 2. Results

### 2.1. Patient Characteristics

In Table 1, the baseline demographics and clinical characteristics of 62 patients with IC/BPS are compared between the Mix and Control groups, including 51 female and 11 male patients (female/male = 5:1), with a median age of 47 [26, 78] years. The median daytime frequency of urination and nocturia per 24 h were 25 [13, 40] and 5 [1, 13] times, respectively. Moreover, the participants’ voided volume per micturition and functional bladder capacity were apparently decreased from normal individuals. Further, there were no significant differences in baseline characteristics between the Mix and Control groups.

### 2.2. Efficacy Assessment

In Table 2, the overall GRA showed that at 3 months post-injection, 19 (67.9%) participants in the Control group and 23 (67.6%) participants in the Mix group had a significant response, without a statistical difference between the two groups. At 6 months, the response rate in the Mix group was remarkably higher than the rate in the Control group (58.8% vs. 28.6%, χ^2^ = 5.7, *p* = 0.02 < 0.05).

In Table 3, at 3 months post-injection, the clinical characteristics in each group had significantly improved from baseline and there was no statistical difference between the two groups. At 6 months, patients’ QoL, VAS, ICSI and ICPI in the Control group had statistically improved from baseline, except for urinary frequency, bladder voided volume and PUF. In the Mix group, patients’ urinary frequency, bladder voided volume, QoL, VAS, PUF, ICSI and ICPI had significantly improved from baseline, with a statistical difference from the Control group.

**Figure 1:**
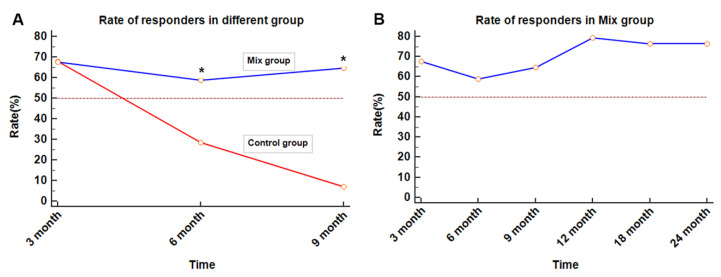
(**A**) * *p* < 0.05, the rates of the responders in the Mix therapy group are significantly higher than those in the Control group after 6 months of treatment. (**B**) In the Mix group, more than half of the participants see a significant improvement within 6 months and a marked improvement at 12 months of treatment. Furthermore, with repeated intravesical instillations of Sapylin, the effect is maintained during the treatment.

The mean effective duration of the responders in the Mix group was apparently better than that in the Control group (27.5 (range 0–89) vs. 4.9 (range 0–11) months, *p* < 0.05). The rates of the responders in the Mix therapy group were significantly higher than those in the Control group after 6 months of treatment (Figure 1A). In the Mix group, more than half of the participants could see a significant improvement within 6 months and a marked improvement at 12 months of treatment (Figure 1B). Furthermore, with repeated intravesical instillations of Sapylin, the great effect continues to maintain during the treatment. With a median follow-up of 32.5 [24.0, 92.0] months, the effective duration of the best responder in the Mix group was 89 months, which continues to last (symptom free).

### 2.3. Safety Assessment

None of the patients experienced serious adverse events. In the Mix group, mild or moderate adverse events related to the BoNT/A injection and Sapylin instillations occurred in 19 (19/34, 55.9%) cases, including 2 cases of acute urinary retention, 10 cases of dysuria, 3 cases of mild hematuria, 3 cases of urinary tract infection and 1 case of mild fever. In the Control group, mild or moderate adverse events related to BoNT/A injection occurred in 13 cases (13/28, 46.4%), including 1 case of acute urinary retention, 8 cases of dysuria, 2 cases of mild hematuria and 2 cases of urinary tract infection. The difference in the adverse events between the two groups had no statistical significance (*p* > 0.05) and they were cured spontaneously without any interventions, or with appropriate treatment, such as antibiotics.

## 3. Discussion

This long follow-up pilot study demonstrated that repeated intravesical instillations of Sapylin after BoNT/A injection could remarkably improve lower urinary tract symptoms and increase the bladder voided volume in IC/BPS patients with tolerable safety, for a long-term effective duration, which was apparently better than BoNT/A injection alone.

It is proposed that IC/BPS might be induced by the interaction among nervous, immune and endocrine factors [1]. The glycosaminoglycan layer protects the bladder mucosa as a chemical barrier against urine. When this layer is defective, it cannot protect the bladder mucosa from infiltrated urine that would induce submucosal inflammation, stimulate persistent sensory nerve hyperactivity and upregulate the urothelium permeability, contributing to urinary frequency and pain [3,4,20].

Previous studies demonstrated that BoNT/A was an effective therapy for IC/BPS; it was recommended by the American Urological Association [21] and the East Asian Urological Association [1]. BoNT/A can inhibit the release of neurotransmitters and neuropeptides (nerve growth factor, calcitonin gene-related peptide and substance P et al.) that regulate pain and inflammation from nerve fibers in the bladder wall and the urothelium to reduce neurogenic inflammation, alleviate neural hypersensitivity and inhibit bladder muscle contraction, which finally improves lower urinary tract symptoms [6]. In 2004, Smith et al. [22] studied 13 IC/BPS patients injected with BoNT/A into the trigone and bladder base, resulting in 69% of the patients seeing a significant improvement in pain. Later, Giannantoni et al. submucosally injected 200 U of BoNT/A into the trigone and bladder floor of 14 patients with IC/BPS. Among them, 12 patients (85.7%) reported subjective improvement at the 1- and 3-month follow-ups; however, the duration only lasted 3 months [23]. In the same group of patients, 13 were followed up with repeated BoNT/A injections for 2 years. A mean of 4.8 ± 0.8 injections were administered per patient, and the mean interval between two consecutive injections was 5.25 ± 0.75 months [24]. Another study reported that at the 5-month follow-up, the beneficial effects persisted in 26.6% of cases and at 12 months after treatment, pain recurred in all the patients [25]. These clinical studies show that the therapeutic duration of BoNT/A in IC/BPS patients is around 3–6 months. Similarly, our present study showed that the response rates of those participants who accepted only a BoNT/A submucosal injection at 3, 6, 9 and 12 months post-injection were 67.9%, 28.6%, 7.1% and 0%, respectively. Interestingly, a randomized comparative study enrolled 34 patients with refractory IC/BPS who were injected with 100 U BoNT/A, mainly into the suburothelial layer [26]. The response rate was 73.5% at 1 month, 58.8% at 3 months, 38.2% at 6 months and 20.6% at 12 months. However, in that study, patients who reported “slightly improved,” “improved” or “remarkably improved,” were considered as the responders to treatment. Further, the treated population might also differ between that study and ours.

Immunotherapy is a reasonable option for IC/BPS [27]. Many researchers found that Sapylin also had immunotherapeutic effects on bladder cancer by initiating marked lymphocytic infiltration around the tumor cells and inhibiting their growth [16,18]. These results suggested that Sapylin might regulate bladder immune and inflammatory responses. In the present study, our results also demonstrated that the response duration of those who received BoNT/A with Sapylin was 27.5 months on average. This long-lasting effect might be attributable mainly to the repeated intravesical treatment of Sapylin. The mechanisms of Sapylin in the treatment of IC/BPS, however, were not investigated in this study. Interestingly, Kong et al. [28] demonstrated that Sapylin could stimulate the body to secrete a variety of cytokines to accelerate wound healing by promoting endothelial cell proliferation, migration and angiogenesis and increasing fibroblast migration and collagen deposition. These results are similar to an animal study [19]. These promising discoveries have encouraged us to explore whether Sapylin could induce immune responses in the bladder to stimulate the proliferation of bladder epithelial cells and inhibit the expression of those inflammatory factors initiated by infiltrated urine.

Our study has several limitations. This was a retrospective, preliminary and single-center pilot study. Although the present study has biases, it was designed as a pilot study to confirm a novel combined therapy for IC/BPS patients. Moreover, we did not directly compare BoNT/A plus Sapylin with other traditional treatments for IC/BPS. Moreover, post-void residual urine and other side effects need to be investigated comprehensively. Finally, we failed to compare the therapeutic effect between BoNT/A plus Sapylin and Sapylin alone. Further research comparing BoNT/A injection plus Sapylin instillation, BoNT/A alone, or Sapylin alone in the treatment of IC/BPS is warranted with a large, multicenter, randomized, placebo-controlled trial.

## 4. Conclusions

This long-follow-up pilot study shows that repeated intravesical instillations of Sapylin after BoNT/A injection can produce significantly better clinical outcomes than BoNT/A alone in IC/PBS patients. Further research comparing BoNT/A injection plus Sapylin instillation, BoNT/A alone, or Sapylin alone in the treatment of IC/BPS is warranted with a large, multicenter, randomized, placebo-controlled trial.

## 5. Materials and Methods

### 5.1. Participants and Ethics

From March 2015 to April 2020 in our hospital, 70 IC/BPS patients were selected according to the following: (1) diagnosed according to National Institute of Diabetes and Digestive and Kidney (NIDDK) guidelines [29]; (2) an O’Leary-Sant Interstitial Cystitis Symptom Index score (ICSI) of more than 9 and disease duration of more than 6 months; (3) previous adequate unsuccessful treatments in accordance with guidelines including oral anti-cholinergic agents, pain-killers, amitriptyline, intravesical hyaluronic acid, heparinoids and so on. Those patients with urinary cancers, bacterial genitourinary infections, bladder tuberculosis, radiation-induced or chemical cystitis, sexually transmitted diseases, pelvic inflammatory disease and endometriosis were excluded [30]. All patients were required to provide their informed consent to receive BoNT/A injection alone or BoNT/A plus Sapylin and they made their own decision about which treatment to receive. Furthermore, 8 cases were removed because of missing data. Finally, this study investigated 34 patients who received repeated intravesical instillations of Sapylin after BoNT/A injection (Mix group) and 28 patients who accepted a submucosal injection of BoNT/A alone (Control group).

After treatment, to evaluate its efficacy, we performed a questionnaire investigation of symptoms via telephone and outpatient interviews. The primary outcome was the global response assessment (GRA) at 3 and 6 months after treatment: “As compared to when you started the current study, how would you rate your overall pelvic symptoms now?”, with seven response categories: 1 = Markedly worse, 2 = Moderately worse, 3 = Slightly worse, 4 = No change, 5 = Slightly improved, 6 = Moderately improved and 7 = Markedly improved. Participants who reported “moderately or markedly improved” were considered as the responders to the treatment. Others were defined as non-responders. The secondary outcomes were pelvic pain and urgency/frequency patient symptom score (PUF), visual analogue scale (VAS), O’Leary-Sant ICSI and Interstitial Cystitis Problem Index (ICPI). Further, a 3-day urinary diary was used to evaluate the mean voided volume per micturition, maximal voided volume per micturition and mean urinary frequency per 24 h. Cystoscopy was performed under local anesthesia before treatment. Saline was delivered into the bladder until the patients’ bladder pain or discomfort became intolerable. Then, the total saline delivery volume was calculated as the functional maximal bladder capacity.

### 5.2. Intervention

#### 5.2.1. Submucosal Injection of BoNT/A

All the patients received a submucosal injection of BoNT/A (Lanzhou Biotechnology Development Co., Ltd., Lanzhou, China) under continuous epidural anesthesia. An amount of 200 U of BoNT/A was diluted in 20 mL of sterile saline in advance, resulting in 10 U of BoNT/A per 1.0 mL. The submucosal injection areas included the left, right, anterior and posterior bladder walls, sparing the trigone. Each of these four bladder walls was injected six times with 8 U of BoNT/A per injection. As dysuria was a common complication and urinary retention was a severe complication, catheterization was routinely applied and removed about 5 days after the operation. An oral antibiotic agent was prescribed for 7 days. Afterwards, the patients in the Mix group accepted intravesical instillation of Sapylin.

#### 5.2.2. Intravesical Instillation of Sapylin

One week after the operation, all patients in the Mix group began to receive intravesical instillation of Sapylin (Sinopharm Group Luya (Shandong) Pharmaceutical Co., Ltd., Jining, China). Sapylin 5 KE (a unit of measure) was evenly dissolved in 40 mL of sterile saline. Mixed Sapylin was instilled into the bladder through a urinary catheter, which was removed after injection, and the Sapylin remained in the bladder until the occurrence of pelvic pain or discomfort but for no more than 2 h. It was instilled once a week within 6 weeks post-injection, then, once every 4 weeks for 2 years, totaling 29 intravesical doses.

### 5.3. Statistical Methods

The continuous variables are expressed as the mean value (standard deviation) and median [Min, Max] and the differences among groups were compared by Student’s t-tests (normal distribution) or Mann–Whitney U test (non-normal distribution). In addition, categorical variables are represented as counts or percentages and the inter-block comparison was analyzed with a chi-squared test. A *p* value of <0.05 was considered statistically significant. SPSS version 26.0 (IBM, Armonk, NY) and MedCalc (MedCalc Software bvba) were used for the statistical analysis.

## Figures and Tables

**Table 1:** Baseline characteristics of the participants.

	Control (*N* = 28)	Mix (*N* = 34)	*p*-Value
Gender			
Male	5 (17.9%)	6 (17.6%)	1
Female	23 (82.1%)	28 (82.4%)	
Age (years)			
Mean (SD)	45.0 (12.5)	46.9 (14.2)	0.569
Median [Min, Max]	41.5 [26.0, 78.0]	48.5 [27.0, 72.0]	
BMI (kg/m^2^)			
Mean (SD)	21.6 (3.5)	21.4 (3.4)	0.835
Median [Min, Max]	21.4 [16.0, 32.0]	20.8 [15.8, 28.2]	
Daytime frequency			
Mean (SD)	26.2 (6.8)	24.8 (7.1)	0.437
Median [Min, Max]	25.0 [13.0, 40.0]	23.0 [14.0, 40.0]	
Nocturia			
Mean (SD)	5.6 (3.4)	5.5 (3.1)	0.909
Median [Min, Max]	5.0 [1.0, 13.0]	5.0 [1.0, 12.0]	
Mean voided volume per micturition (mL)			
Mean (SD)	56.2 (22.6)	57.1 (24.0)	0.883
Median [Min, Max]	54.0 [23.0, 125.0]	54.0 [22.0, 110.0]	
Maximal voided volume per micturition (mL)			
Mean (SD)	88.9 (26.6)	86.5 (25.2)	0.804
Median [Min, Max]	83.0 [57.0, 163.0]	83.5 [51.0, 152.0]	
Functional bladder capacity (mL)			
Mean (SD)	152.0 (49.3)	146.0 (48.8)	0.561
Median [Min, Max]	143.0 [90.0, 260.0]	138.0 [90.0, 255.0]	
QoL			
Mean (SD)	5.8 (0.5)	5.7 (0.4)	0.906
Median [Min, Max]	6.0 [4.0, 6.0]	6.0 [5.0, 6.0]	
VAS			
Mean (SD)	8.8 (1.3)	8.8 (1.3)	0.964
Median [Min, Max]	9.0 [6.0, 10.0]	9.0 [6.0, 10.0]	
PUF			
Mean (SD)	24.3 (4.4)	24.4 (4.6)	0.738
Median [Min, Max]	24.5 [15.0, 31.0]	25.0 [15.0, 31.0]	
ICSI			
Mean (SD)	16.1 (2.7)	16.1 (2.7)	0.971
Median [Min, Max]	16.0 [10.0, 20.0]	16.5 [10.0, 20.0]	
ICPI			
Mean (SD)	14.8 (1.5)	14.8 (1.6)	0.725
Median [Min, Max]	15.0 [9.0, 16.0]	15.5 [10.0, 16.0]	

BMI: body mass index; QoL: quality of life; VAS: visual analogue scale; PUF: pelvic pain and urgency/frequency patient symptom score; ICSI: O’Leary-Sant Interstitial Cystitis Symptom Index; ICPI: O’Leary-Sant Interstitial Cystitis Problem Index.

**Table 2:** Global response assessment at 3 and 6 months.

	Control (*N* = 28)	Mix (*N* = 34)	*p*-Value
3 Months			
7 = Markedly improved	2 (7.1%)	2 (5.9%)	
6 = Moderately improved	17 (60.7%)	21 (61.8%)	
5 = Slightly improved	4 (14.3%)	6 (17.6%)	
4 = No change	5 (17.9%)	5 (14.7%)	
3 = Slightly worse	0 (0.0%)	0 (0.0%)	
2 = Moderately worse	0 (0.0%)	0 (0.0%)	
1 = Markedly worse	0 (0.0%)	0 (0.0%)	
Non-responders (1 + 2 + 3 + 4 + 5)	9 (32.1%)	11 (32.4%)	1
Responders (6 + 7)	19 (67.9%)	23 (67.6%)	
6 Months			
7 = Markedly improved	2 (7.1%)	4 (11.8%)	
6 = Moderately improved	6 (21.4%)	16 (47.1%)	
5 = Slightly improved	6 (21.4%)	11 (32.4%)	
4 = No change	9 (32.1%)	3 (8.8%)	
3 = Slightly worse	5 (17.9%)	0 (0.0%)	
2 = Moderately worse	0 (0.0%)	0 (0.0%)	
1 = Markedly worse	0 (0.0%)	0 (0.0%)	
Non-responders (1 + 2 + 3 + 4 + 5)	20 (71.4%)	14 (41.2%)	<0.05
Responders (6 + 7)	8 (28.6%)	20 (58.8%)	

**Table 3:** Secondary outcome changes from baseline to 3 and 6 months after treatment.

	Baseline		3 Months		6 Months	
	Control (*N* = 28)	Mix (*N* = 34)	Control (*N* = 28)	Mix (*N* = 34)	Control (*N* = 28)	Mix (*N* = 34)
Daytime frequency						
Mean (SD)	26.2 (6.8)	24.8 (7.1)	13.5 (7.8) ^b^	12.5 (6.7) ^b^	21.5 (10.1)	11.7 (5.3) ^a, b^
Median [Min, Max]	25.0 [13.0, 40.0]	23.0 [14.0, 40.0]	10.0 [6.0, 35.0]	10.0 [5.0, 33.0]	24.0 [6.0, 35.0]	10.0 [4.0, 26.0]
Nocturia						
Mean (SD)	5.6 (3.4)	5.5 (3.1)	2.3 (2.1) ^b^	2.3 (2.1) ^b^	4.1 (3.2)	2.3 (1.7) ^a,b^
Median [Min, Max]	5.0 [1.0, 13.0]	5.0 [1.0, 12.0]	2.0 [0.0, 9.0]	2.0 [0.0, 9.0]	3.5 [0.0, 11.0]	2.0 [0.0, 7.0]
Mean voided volume per micturition (mL)						
Mean (SD)	56.2 (22.6)	57.1 (24.0)	138.0 (69.9) ^b^	144.0 (79.8) ^b^	92.1 (73.5)	152.0 (91.7) ^a,b^
Median [Min, Max]	54.0 [23.0, 125.0]	54.0 [22.0, 110.0]	143.0 [28.0, 301.0]	135.0 [34.0, 402.0]	59.0 [27.0, 289.0]	128.0 [39.0, 395.0]
Maximal voided volume per micturition (mL)						
Mean (SD)	88.9 (26.6)	86.5 (25.2)	171.0 (71.2) ^b^	178.0 (79.7) ^b^	120.0 (75.2)	188.0 (99.3) ^a,b^
Median [Min, Max]	83.0 [57.0, 163.0]	83.5 [51.0, 152.0]	174.0 [63.0, 338.0]	170.0 [61.0, 438.0]	90.0 [56.0, 321.0]	174.0 [59.0, 432.0]
QoL						
Mean (SD)	5.8 (0.5)	5.7 (0.4)	2.7 (1.9) ^b^	2.9 (2.0) ^b^	4.4 (2.1) ^b^	2.9 (1.9) ^a,b^
Median [Min, Max]	6.0 [4.0, 6.0]	6.0 [5.0, 6.0]	2.0 [0.0, 6.0]	2.0 [0.0, 6.0]	5.0 [0.0, 6.0]	2.5 [0.0, 6.0]
VAS						
Mean (SD)	8.8 (1.3)	8.8 (1.3)	4.9 (2.2) ^b^	5.1 (2.3) ^b^	7.0 (3.1) ^b^	4.9 (2.4) ^a,b^
Median [Min, Max]	9.0 [6.0, 10.0]	9.0 [6.0, 10.0]	4.0 [1.0, 10.0]	4.5 [1.0, 10.0]	8.0 [1.0, 10.0]	5.0 [0.0, 9.0]
PUF						
Mean (SD)	24.3 (4.4)	24.4 (4.6)	13.8 (5.3) ^b^	14.2 (5.6) ^b^	20.1 (7.5)	14.0 (5.4) ^a,b^
Median [Min, Max]	24.5 [15.0, 31.0]	25.0 [15.0, 31.0]	12.0 [6.0, 29.0]	12.0 [8.0, 28.0]	21.5 [8.0, 31.0]	12.5 [6.0, 26.0]
ICSI						
Mean (SD)	16.1 (2.7)	16.1 (2.7)	9.7 (3.5) ^b^	9.7 (3.3) ^b^	13.6 (4.5) ^b^	9.5 (3.0) ^a,b^
Median [Min, Max]	16.0 [10.0, 20.0]	16.5 [10.0, 20.0]	8.50 [3.0, 19.0]	8.0 [6.0, 19.0]	14.0 [6.0, 20.0]	9.5 [5.0, 16.0]
ICPI						
Mean (SD)	14.8 (1.5)	14.8 (1.6)	9.0 (3.0) ^b^	9.2 (2.8) ^b^	12.4 (3.8) ^b^	8.9 (2.7) ^a,b^
Median [Min, Max]	15.0 [9.0, 16.0]	15.5 [10.0, 16.0]	8.0 [4.0, 16.0]	9.0 [6.0, 15.0]	14.0 [5.0, 16.0]	9.0 [4.0, 14.0]

QoL: quality of life; VAS: visual analogue scale; PUF: pelvic pain and urgency/frequency patient symptom score; ICSI: O’Leary-Sant Interstitial Cystitis Symptom Index; ICPI: O’Leary-Sant Interstitial Cystitis Problem Index. ^a^ *p* < 0.05: Mix group compared to Control group. ^b^ *p* < 0.05: Mix or Control group compared to corresponding baseline.

## Data Availability

The data presented in this study are available on request from the corresponding author. The data are not publicly available.
